# The United States Withdrawal From the World Health Organization: Implications and Challenges

**DOI:** 10.34172/ijhpm.9086

**Published:** 2025-03-18

**Authors:** Vahid Yazdi-Feyzabadi, Ali-Akbar Haghdoost, Martin McKee, Amirhossein Takian, Elizabeth Bradley, Ruairí Brugha, Nir Eyal, Sana Eybpoosh, Lawrence Gostin, Naoki Ikegami, Ilona Kickbusch, Ronald Labonté, Russell Mannion, Ole F. Norheim, Jeremy Shiffman, Mohammad Karamouzian

**Affiliations:** ^1^Health Services Management Research Center, Institute for Futures Studies in Health, Kerman University of Medical Sciences, Kerman, Iran.; ^2^Modeling in Health Research Center, Institute for Future Studies in Health, Kerman University of Medical Sciences, Kerman, Iran.; ^3^Centre for Global Chronic Conditions, London School of Hygiene and Tropical Medicine, London, UK.; ^4^Centre of Excellence for Global Health, Department of Global Health & Public Policy, School of Public Health, Tehran University of Medical Sciences (TUMS), Tehran, Iran.; ^5^Vassar College, Poughkeepsie, NY, USA.; ^6^Department of Public Health and Epidemiology, RCSI University of Medicine and Health Sciences, Dublin 2, Ireland.; ^7^School of Public Health, Rutgers, The State University of New Jersey, Piscataway, NJ, USA.; ^8^Institute for Health, Rutgers University, New Brunswick, NJ, USA.; ^9^Department of Epidemiology and Biostatistics, Research Centre for Emerging and Reemerging Infectious Diseases, Pasteur Institute of Iran, Tehran, Iran.; ^10^O’Neill Institute for National and Global Health Law, Georgetown University Law Center, Washington, DC, USA.; ^11^Keio University, Tokyo, Japan.; ^12^Graduate Institute for International and Development Studies, Geneva, Switzerland.; ^13^School of Epidemiology, Public Health and Preventive Medicine, University of Ottawa, Ottawa, ON, Canada.; ^14^Health Services Management Centre, University of Birmingham, Birmingham, UK.; ^15^Department of Global Public Health and Primary Care, University of Bergen, Bergen, Norway.; ^16^Paul H Nitze School of Advanced International Studies, Johns Hopkins University, Baltimore, MD, USA.; ^17^Centre on Drug Policy Evaluation, MAP Centre for Urban Health Solutions, St. Michael’s Hospital, Toronto, ON, Canada.; ^18^Dalla Lana School of Public Health, University of Toronto, Toronto, ON, Canada.; ^19^HIV/STI Surveillance Research Center, and WHO Collaborating Center for HIV Surveillance, Institute for Futures Studies in Health, Kerman University of Medical Sciences, Kerman, Iran.

**Keywords:** Global Health, Nationalism, Political Determinants of Health, United States of America, World Health Organization

## Abstract

President Trump’s 2025 decision to remove the United States (US) from the World Health Organization (WHO), echoing his initial 2020 move, raises existential questions about the future of global health governance. This editorial explores the immediate and long-term potential impacts of the withdrawal, noting that it poses a significant threat to the WHO financing. This, in turn, will have adverse consequences for future pandemic preparedness, health inequities, and cross-border collaboration. We also explore the potential role of private philanthropies in bridging the funding gap, against the risk of shifting health priorities away from local needs. For the US, withdrawal means diminished influence on global health policies and weaker alignment with new international regulations. Moving forward, structural reforms within the WHO, equitable contributions from global powers, and renewed US involvement are essential to maintain strong health systems worldwide. Ultimately, a collaborative approach is necessary to uphold collective preparedness against emerging health crises.

## Introduction

 The World Health Organization (WHO) is the specialized United Nations agency dedicated to health. Through meaningful cooperation between the secretariat and 194 member states, the WHO coordinates global efforts to define norms and regulatory standards, address health crises, combat diseases, and strengthen health systems.^1^ During the COVID-19 pandemic, the WHO’s guidance on vaccine distribution and public health measures demonstrated its unparalleled leading role in coordinating global responses.^2^ In an era defined by complex health threats—ranging from antimicrobial resistance to climate-related health risks—the WHO’s convening power remains critical for forging partnerships, sharing knowledge, and implementing effective, equitable health interventions to reach sustainable health development everywhere.

 On January 20, 2025, President Donald Trump announced that the United States (US) would withdraw from the WHO, echoing the move he implemented in July 2020,^3^ which was reversed by his successor, President Biden, in 2021. The new announcement has reignited concerns over its far-reaching consequences.^4-6^ As one of the WHO’s major financial contributors,^7^ the US’s role in sustaining the organization’s programs is significant. The US is already disengaging, preventing Centers for Disease Control and Prevention (CDC) employees from co-authoring papers with the WHO staff,^8^ disrupting mechanisms for addressing transnational health threats, and weakening the WHO’s financial stability and operational capacity, particularly in low- and middle-income countries (LMICs).^9,10^ The 2025 withdrawal comes amid a highly strained global health landscape, jeopardizing the slow progress in pandemic preparedness, health equity, and prevention and control of non-communicable diseases. This editorial assesses the potential impact of the withdrawal on global health governance, focusing on trends in US foreign policy and the implications for multilateral health initiatives in an interconnected world.

## The US as Founder, Major Funder, and the First to Withdraw

 The US was instrumental in establishing the WHO in 1948, recognizing the need for a unified international body to combat global health threats.^11^ Over several decades, the US became both a principal funder and a major driver of the WHO’s agenda, even as it levied criticism at times against the organization’s governance and effectiveness.^10^ This contradiction underscores the complexity of the US’s role in international health governance; on one hand, it has led significant multilateral initiatives, and on the other, it has never hesitated to question or withdraw support when political priorities shift. In practical terms, however, the US role has been uniquely influential due to the interplay of assessed and voluntary contributions. In 2022–2023, the US contributed around US$218 million in assessed contributions, plus another US$1.065 billion in voluntary, largely earmarked funds.^7,12^ Moreover, American scientists, institutions, and public health experts have been among key players in shaping the WHO policies, advancing research, and guiding responses to outbreaks such as COVID-19—reinforcing the US’s position as a global public health partner.^13^

 Historically, the US contributions have driven key global health efforts, including vaccine research, disease eradication campaigns, and pandemic preparedness initiatives. One of the most notable examples is the President’s Emergency Plan For AIDS Relief (PEPFAR). Introduced under President George W. Bush, PEPFAR has saved millions of lives by addressing the HIV/AIDS crisis through both bilateral and multilateral collaboration—demonstrating that bipartisan support for large-scale global health programs is possible, even under Republican leadership.^14,15^ However, the US withdrawal from the WHO in 2025 represents a stark departure from this legacy. The first Trump administration’s 2020 announcement alone reduced the WHO funding by an estimated US$400 million,^9^ disrupting vaccination campaigns and disease-control projects in LMICs. Now, in an even more strained global health landscape, the second Trump administration claims the WHO is hampered by mismanagement, corruption, and unfair financial burdens on the US, especially compared to China. The administration cites China’s low voluntary contribution (~US$42 million) as evidence of an imbalance,^4^ even though the US’s “modest” 20% share of the WHO budget is still proportionate to just a fraction of its approximate 25% share of global gross domestic product (GDP).^16,17^ Some argue this contribution could be increased rather than reduced,^18^ while calling upon China to contribute more to the WHO budget.^10^

 Beyond concerns about the WHO’s financial stability and fears of eroding US leadership, the vacuum left by reduced federal US funding could potentially be addressed in part by private philanthropists. The Bill & Melinda Gates Foundation ranks just behind the US as the WHO’s second-largest voluntary funder.^19^ Despite its potential benefits, such “philanthro-capitalist” models have raised concerns about limited transparency, less inclusive governance, and potential conflicts of interest. Critics warn that large-scale private contributions might shift global health decision-making to a small group of wealthy donors, favoring top-down solutions that may not fully address the needs and preferences of LMICs.^20-22^ Furthermore, certain philanthropic interventions risk commercializing patient care or diverting investment away from strengthening public health systems.^23^ As the WHO navigates these shifts, uncertainty grows regarding who will finance essential health programs and how these new funding patterns will influence governance structures.

 Ultimately, the US withdrawal calls into question the long-term stability of the WHO’s funding model and highlights broader power imbalances in global health. While it is clear that some major economies under-contribute relative to their GDP, the US’s stance now raises profound concerns about whether national interests might supersede collective efforts to safeguard global health. Although this decision aligns with an administration that has shown contempt for multilateral governance, it stands at odds with past Republican-led global health success stories, such as PEPFAR. The move, therefore, underscores not only a shift in US foreign policy but also the fragility of international health organizations that rely on consistent, collaborative support from their most capable members.

## Domestic and Global Consequences of US’s Withdrawal From the WHO

 By exiting the WHO, the US severs ties with a crucial multilateral framework and forfeits influence in revising the International Health Regulations and shaping the Pandemic Accord, both essential for enhancing global preparedness and coordinated response to emerging health threats. This retreat, however, aligns with right-wing populist movements, such as the current Argentinian government, that favor unilateral approaches, questioning the legitimacy of multilateral institutions.^24,25^ Domestically, losing the WHO membership decreases real-time access to disease surveillance, technical guidance, and early warning networks—resources that proved essential during COVID-19.^26,27^ Institutions, such as the CDC and National Institutes of Health (NIH), already weakened by the administration’s political attacks,^28,29^ lose collaborative opportunities to shape global health research priorities. One example is the funding freeze on the United States Agency for International Development (USAID) Malaria Vaccine Development Program, which delayed crucial vaccine trials and set back years of progress.^30^ The weakened infrastructure leaves the US more exposed to emergent threats, such as avian influenza, by slowing detection and response efforts.

 Globally, the withdrawal would create a budget deficit at the WHO, disrupting vital programs, such as vaccination campaigns, maternal and child health, and health emergency preparedness in LMICs. These initiatives serve as lifelines for vulnerable populations toward reducing health inequities through the Sustainable Development Goals.^31^ Moreover, focusing on short-term financial gains overlooks how weakened WHO-led initiatives can drive cross-border health risks—from antibiotic-resistant pathogens to newly emerging viruses—that may inevitably threaten US citizens.

 Beyond immediate funding gaps, the US departure signals to other member states that populist or nationalist agendas may supersede multilateral action. This would shrink the WHO’s capacity to coordinate on increasingly pressing health issues. The health of the global population depends on preventing major funders from following the US’s lead in cutting the WHO budget and reducing its unique role during health crises. Over time, the combined effects of diminished funding, fragmented commitments, and weakened leadership undermine global health governance. In an era in which transnational health challenges require united solutions, the US withdrawal risks amplifying health threats worldwide, while simultaneously eroding its own health security and global standing (Table).

**Table:** Potential Implications of US Withdrawal from the WHO

**Scope**	**Negative Consequences**	**Positive Consequences**
For Global Health Arena	☒ Funding gaps for global health programs, particularly those in LMICs ☒ Reduced US influence in shaping global health policies ☒ Increasing global health inequities ☒ Reduced collaboration in pandemics ☒ Erosion of global community trust in countries’ commitments, particularly the US’s	☑ "Wake-up call" or a similar effect that drives long-term capacity building for multilateral collaborations
For the US	☒ Loss of technical guidance from the WHO ☒ Disruption in access to global health resources such as the WHO’s disease surveillance system ☒ Erosion of US global health leadership and weakening US-led global health initiatives ☒ Long-term health crises returning to impact the US due to lack of international collaboration ☒ Strained relationships with allied countries that prioritize multilateral cooperation	☑ Short-term and minimal financial savings (0.003% of US GDP) ☑ Reduced US financial burden through multilateral systems ☑ Potential redirection of funding to domestic issues

Abbreviations: WHO, World Health Organization; LMICs, low- and middle-income countries; GDP, gross domestic product.

## Recommendations and Ways Forward

 Immediate action is needed to contain the damage caused by the US withdrawal from the WHO and to preserve the WHO’s capacity to coordinate global health efforts effectively. First, we advocate for member states and partners to pursue governance reforms to expand and diversify the financial base of the WHO, aiming to reduce its reliance on any single major contributor.

 Second, we propose acknowledging the WHO’s less visible but equally critical functions while prioritizing necessary reforms. Beyond its public-facing role in crisis response, the WHO’s behind-the-scenes work includes comprehensive global health surveillance, maintaining vital supply chains for vaccines and medications that benefit both recipient nations and American businesses, and providing specialized technical assistance that strengthens health systems worldwide. These contributions represent a significant return on investment for member states. Despite these great contributions, enhancing transparency, streamlining decision-making processes, and implementing recommendations from the Independent Panel on Pandemic Preparedness and Response is needed, and would significantly improve the organization’s effectiveness. Greater involvement from emerging economies and non-state actors would revitalize global engagement while addressing legitimate concerns about representation. These targeted reforms would strengthen the WHO’s credibility while encouraging high-income countries to increase their financial commitments rather than retreat from this irreplaceable institution.

 Lastly, restoring the US’s role within the WHO is vital for collective health security. Although current domestic political conditions prevent cooperation, a strategic emphasis on national interests, (ie, early-warning systems, real-time disease surveillance, and targeted research collaborations)could gradually rebuild bipartisan support in the US Congress. Diplomatic engagement and advocacy by stakeholders both inside and outside the US may lay the groundwork for a future re-entry,^32^ especially if political leadership shifts. Ultimately, international solidarity in confronting pandemics and health inequities stands as the most robust safeguard for global stability and prosperity, underscoring the urgent need to keep multinational partnerships strong, even when a major player steps away.

## Epilogue

 While the WHO remains a central pillar of global health governance, the US withdrawal from the WHO highlights the need to prepare for uncertainties threatening global health security and the repercussions of unilateral actions by major funders. The US decision to step away underscores a broader shift toward nationalist policies that can undermine collective preparedness for pandemics and other crises. Greater financial investment in the WHO—coupled with robust reforms to improve transparency and effectiveness—would help mitigate these vulnerabilities and reinforce a united front against emerging threats. The international community must reaffirm its commitment to multilateralism while encouraging wealthier nations to shoulder their fair share of responsibility. The US, in particular, should reassess its stance, as its engagement remains critical for shaping international responses and driving equitable health outcomes. While withdrawing from global institutions may offer temporary political advantages, in the long run, it leaves the US more susceptible to public health emergencies that demand concerted, transnational efforts. In our interconnected world, no population is secure until all are protected. The success of any global health strategy rests on sustained cooperation, without which the very mechanisms designed to protect populations worldwide risk becoming fragmented.

## Ethical issues

 Not applicable.

## Conflicts of interest

 Martin McKee is co-Director of the European Observatory on Health Systems and Policies, a partnership hosted by WHO and an adviser to WHO EURO. The rest of the authors declare that they have no conflicts of interest.
